# CYB5R3: a key player in aerobic metabolism and aging?

**DOI:** 10.18632/aging.100112

**Published:** 2009-12-29

**Authors:** Rafael de Cabo, Emilio Siendones, Robin Minor, Plácido Navas

**Affiliations:** ^1^ Laboratory of Experimental Gerontology, National Institute on Aging, NIH, Baltimore, MD 21224, USA; ^2^ Centro Andaluz de Biología del Desarrollo, Universidad Pablo de Olavide-CSIC and Centre for Biomedical Research on Rare Diseases (CIBERER), ISCIII, E-41013 Sevilla, Spain

**Keywords:** cytochrome b, reductase, NAD, /NADH, NQR1, lifespan, respiration

## Abstract

Aging results from a complex and not completely understood chain of processes that are associated with various negative metabolic consequences and ultimately leads to senescence and death. The intracellular ratio of pyridine nucleotides (NAD^+^/NADH), has been proposed to be at the center stage of age-related biochemical changes in organisms, and may help to explain the observed influence of calorie restriction and energy-sensitive proteins on lifespan in model organisms. Indeed, the NAD^+^/NADH ratios affect the activity of a number of proteins, including sirtuins, which have gained prominence in the aging field as potential mediators of the beneficial effects of calorie restriction and mediating lifespan. Here we review the activities of a redox enzyme (NQR1 in yeast and CYB5R3 in mammals) that also influences the NAD^+^/NADH ratio and may play a regulatory role that connects aerobic metabolism with aging.

Aging involves multiple processes that render cells, tissues and organs vulnerable to stress, damage and ultimately death. Aging itself is not a disease, but there are a number of diseases that become exponentially more prevalent with advancing age such as cancer, cardiovascular disease, metabolic syndrome and neurodegenerative diseases. Energy sensing, food intake and caloric utilization must be kept in equilibrium to preserve appropriate fat stores to prevent the deregulation of glucose homeostasis and other obesity-related disorders.

Calorie restriction (CR) is an intervention aimed to produce undernutrition without malnutrition. CR increases healthspan and lifespan in almost all species tested such as yeast, insects, nematodes and mammals [1], including nonhuman primates [2]. CR has been studied extensively with consistent results showing its beneficial effects on longevity,  age-associated diseases, attenuation of functional decline, and carcinogenesis across a variety of species and diet formulations [3]. Among mammals mice have been the most heavily-researched model with CR eliciting myriad behavioral, physiological, and metabolic changes that include decreased body temperature, blood glucose, insulin and fat mass, and increased physical activity, glucose tolerance and insulin sensitivity [4].  Studies in *Saccharomyces cerevisiae* and *Drosophila melanogaster* have demonstrated that *SIR2*, which encodes for a NAD^+^-dependent histone deacetylase, plays a central role in mediating the increase in longevity associated with CR in these species [5-7]. The involvement of *SIR2* in lifespan extension by CR may relate to its responsiveness to nicotinamide levels and the NAD^+^/NADH ratio, both indicators of cellular energy status [8-10]. A growing body of evidence indicates that the mammalian homologue of *SIR2*, *SIRT1*, also plays a significant role in responding to CR. For example, CR elevates *SIRT1* expression in a number of tissues [11], and transgenic mice that overexpress *SIRT1* exhibit a phenotype mirroring some aspects of CR [12]. *SIRT1* has also been shown to improve insulin sensitivity [13], another consequence of a CR diet [14].

CR promotes a healthy aging phenotype through a myriad of mechanisms, one of which is thought to be its ability to increase mitochondrial efficiency and biogenesis. Increases in mitochondrial biogenesis are driven by eNOS and *PGC-1α* expression and activation. Furthermore, these changes in mitochondria following CR are accompanied by a decrease in production of reactive oxygen species (ROS) without a net reduction of ATP biosynthesis, which indicates a higher bioenergetics efficiency [15,16]. There are several reports on how CR induces the deacetylation of *PGC-1α* by *SIRT1*[17]. Sirtuins are NAD^+^-dependent deacetylases [18], and this dependence has led researchers to propose that sirtuins are at the center of the regulatory nexus between energy metabolism and aging because NAD^+^ is a primary marker for intracellular energy status. It has also been demonstrated that CR activates sirtuins and thereby increases both the stability of chromatin [19] and cell survival [11]. Given the dependence of sirtuins on NAD^+^ and the published activities of sirtuins under CR conditions, it has been hypothesized that NAD^+^ levels and its metabolism are at the center of the regulatory mechanisms behind the beneficial effects of CR [17]. Furthermore, the conversion of NADH to its reduced form NAD^+^ in mitochondria, a reaction that is supported by coenzyme Q (CoQ), is also thought to protect mitochondria during aging [20]. We propose, therefore, mechanisms that affect the NAD^+^/NADH ratio and thereby modulate sirtuins and other NAD^+^-dependent enzymes are key players in the regulation of the aging process.

We have recently described the role of *NQR1*, a gene that encodes cytochrome *b_5_* reductase, a protein that uses both NADH and CoQ as substrates, in chronological and replicative lifespan in *Saccharomyces cerevisiae* [21]. This enzyme is located at the plasma membrane and is homologous to the mammalian enzyme encoded by *CYB5R3*, which can also be found in plasma membranes and uses exclusively NADH and CoQ as substrates [22]. This enzyme is a key component of the trans-plasma membrane redox system (PMRS). The PMRS provides both protection against extracellular oxidants [23] and prevention of apoptosis initiated by the activation of the neutral sphingo-myelinase at the plasma membrane [24]. CR induces the expression of *NQR1* in yeast, increasing the cytosolic NADH oxidation rate [21]. Similarly, CR increases the presence of this enzyme in the plasma membranes of both the liver and brain of rats, improving the antioxidant protection of phospholipids in these membranes [25,26]. This antioxidant system is also activated in mitochondrial DNA-deficient (ρ°) mammalian cells [27,28], and in vitamin E-deficient rat livers [29]. In the case of mammalian ρ° cells, cell survival is dependent on the redox homeostasis maintained by NADH oxidation by the PMRS. As indicated above, the increase of aerobic metabolism induced by CR also requires the cytosolic cooperation of *CYB5R3* to maintain the NAD^+^/NADH ratio. Thus, any intervention that induces membrane instability or alters respiratory metabolism will evoke the transcription of *CYB5R3* and activation of its enzymatic product.

Similar to the case in mammals, yeast *NQR1* is upregulated by CR in parallel with an activation of respiration. Given that the same conditions activate the CoQ biosynthesis pathway [30], this may indicate a connection between CoQ biosynthesis and respiration. Interestingly, over-expression of *NQR1* in yeast requires respiration to maintain cell survival. The mitochondrial mutant strains *ΔATP2* and *ΔCOR1* cannot grow under anaerobic conditions when *NQR1* is overexpressed. The *ΔATP2*strain has a defective ATP synthase complex and the *ΔCOR1 *strain is defective in the bc_1 _complex. Similar results are obtained when the *ΔCOQ2* strain, in which the CoQ biosynthesis pathway is inoperable, is used to overexpress *NQR1*. However, the addition of external CoQ_6_ restored both respiration and growth in the latest strain. These results indicate that *NQR1* effect acts through the respiratory metabolism in yeast [21].

Over-expression of *NQR1* extends chronological lifespan in the absence of *SIR2*, perhaps acting through a pathway dependent on NAD^+^/NADH balance that requires respiration [31], but not *SIR2* [32]. *NQR1* over-expression also extends replicative lifespan in a *SIR2-*dependent manner that mimics CR[8]. *NQR1* promotes oxygen consumption while inhibiting ethanol production and this shift occurs alongside an increase in respiratory chain enzyme activities.* NQR1* thus causes a shift from fermentative to respiratory metabolism that may help explain its role in longevity. Yeast growing in low glucose (CR) media also shows the increase of both chronological and replicative lifespan through the activation of respiration [8,31].

We can hypothesize then that *NQR1 *in yeast and* CYB5R3* in mammals play a regulatory role connecting aerobic metabolism and aging processes through their ability to alter the NAD^+^/NADH ratio. Cytosolic NAD^+^/NADH must be balanced with that of mitochondria. We expect that *NQR1* would partially prevent the *de novo* biosynthesis of NAD^+^ most likely by increasing the recycling of the redox state of nucleotides and maintaining the availability of NAD^+^ to consumer enzymes.

It is assumed that sirtuins connect metabolism to aging because they use NAD^+^ as substrate [18]. This rationale can also be applied to *CYB5R3* because the enzyme consumes NADH as an obligatory substrate. This enzyme would then be an essential component of the NAD^+^/NADH-dependent metabolic pathways in cooperation with the mitochondrial respiratory chain (Figure 1), which both contribute to the maintenance of the NAD^+^/NADH ratio and, as a consequence, regulate the function of sirtuins and other downstream NAD^+ ^consumers. The NADH consumers and NAD^+^ consumers may participate in a regulatory loop, as a decrease of NAD^+^ availability will activate NAD^+^ biosynthesis as has been shown to occur under stress such as in nutrient-dependent survival mechanisms [33].

Mammalian *CYB5R3* may also connect aerobic metabolism and aging. *CYB5R3* encodes for a membrane-bound form of cytochrome *b_5_*reductase in somatic cells that is N-myristoylated and thereby anchored to the plasma membrane, mitochondrial outer membrane and endoplasmic reticulum. This isoform participates in cholesterol biosynthesis [34], fatty acid elongation and desaturation [35], P-450 mediated  hydroxylation of drugs and steroid hormones [36] and the PMRS [22]. There is also a soluble isoform, which lacks the N-terminal binding domain and exists in the cytoplasm of erythrocytes where its main function is to reduce methaemoglobin [37]. Both isoforms come from alternative splicing of the same *CYB5R3* gene.Deficiencies of cytochrome *b_5_*reductase cause recessive congenital methaemoglobulinemia (RCM), which presents with two distinct clinical forms. RCM type I is benign and limited to red blood cells. RCM type II is severe, affects all cells in the organism, and can lead to neurological dysfunction (for review see [38]).

**Figure 1.**
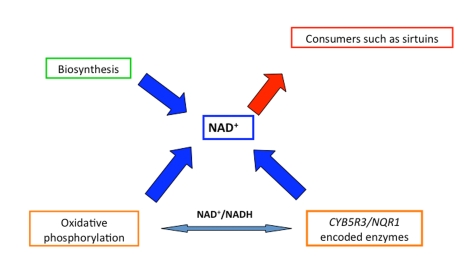
Role of the different characters to guarantee the availability of NAD+ to consumers maintaining at the same time the cellular redox homeostasis through a balanced NAD ^+^/NADH ratio.

Recently, the proteomic profile of metabolic proteins in the invasive glioblastoma phenotype has been studied by applying a functional analysis using the Ingenuity Pathway Knowledge Base (Ingenuity Systems, Redwood City, CA) [39]. The results identified oxidative phosphorylation, mitochondrial dysfunction and ubiquinone biosynthesis as canonical pathways of the cancerous phenotype and *CYB5R3* is identified as a protein associated with the mitochondrial dysfunction pathway. Furthermore, the relationship between mitochondrial dysfunction and *CYB5R3* has also reported in a study carried out to analyze gene expression induced by bromide exposure using the Ingenuity Pathway Analysis[40].

Data from our laboratory seem to indicate a positive role for mammalian *CYB5R3* in mitochondrial respiration. We have used siRNA technology to silence *CYB5R3* in cultured human cells (Figure 2). Preliminary results indicate that *CYB5R3* KO cells exhibit an apparent senescent phenotype based on the accumulation of β-galactosidase. These cells also show a reduction in the mitochondrial respiration rate based on analysis of oxygen consumption. Biochemical analysis of these cells also revealed an increase in the expression of *PGC-1α* that indicates increased recycling or *de novo* biogenesis of mitochondria. In a recently-reported global analysis of lysine-acetylated proteins, a posttranslational modifica-tion of *CYB5R3-*encoded protein by lysine acetylation in its FAD-binding domain has been identified [41]. Lysine acetylation is necessary for the interaction between *SIRT1* and other sirtuins their targets before deacetylation can occur.  Though conclusive experimental data still need to be shown, we hypothesize that *SIRT1* regulates the cytosolic NAD^+^/NADH ratio by influencing *CYB5R3* activity (Figure 3). Conditions of high NADH would lead to partial inactivation of *SIRT1*, leading to an accumulation of the acetylated form of *CYB5R3*. This active form of *CYB5R3* would increase NADH oxidation and release NAD^+^ that, in turn, would activate *SIRT1*. *CYB5R3* would be then deacetylated, causing a decrease in its activity and thereby maintaining the NAD^+^/NADH ratio in proper balance.* PGC-1α* activity will be also affected by this cycle through its interaction with *SIRT1*. Taken together, our preliminary data indicate *CYB5R3* could play an essential role in the mitochondrial metabolism by its contribution to cellular redox homeostasis. A coordination of the redox balance in both the cytosol and mitochondria appears to be necessary for optimum cellular health, and may be of consequence to healthy aging as well.

**Figure 2.**
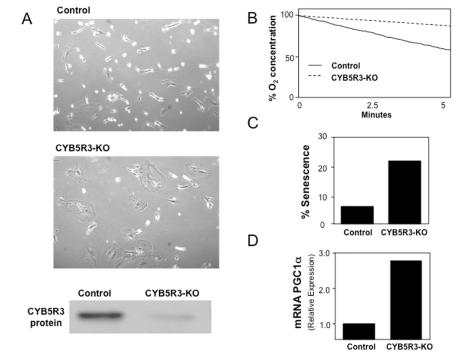
MRC-5 normal human diploid fibroblasts were CYB5R3-silenced (KO cells) and cultured in DMEM medium supplemented with FBS 10%. (**A**) Cell growth and *CYB5R3* protein levels after five days of *CYB5R3*-silencing are shown. (**B**) Oxygen consumption was measured in parallel in both control and *CYB5R3*-KO cells. (**C**) Percentage of senescence was determined by senescence-associated-β-galactosidase activity. (**D**) Total RNA was extracted in both control and *CYB5R3*-KO cells and *PGC1α* mRNA levels were obtained by real time PCR.

**Figure 3.**
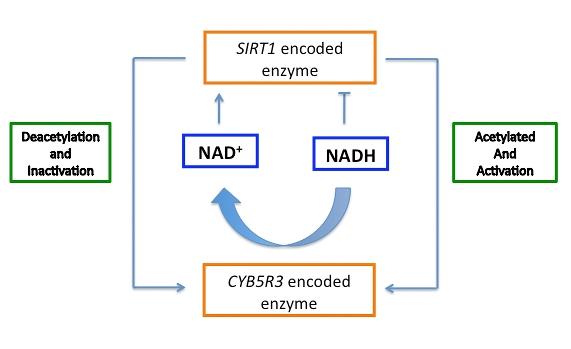
Hypothesis of the regulatory connection between cytochrome *b_5_* reductase and sirtuin to maintain *SIRT1* dependent respiration and cytosolic NAD^+^/NADH ratio.

## References

[1] Bartke A, Wright JC, Mattison JA, Ingram DK, Miller RA, Roth GS (2002). Dietary restriction and life-span. Science.

[2] Mattison JA, Roth GS, Lane MA, Ingram DK (2007). Dietary restriction in aging nonhuman primates. Interdiscip Top Gerontol.

[3] Weindruch R, Sohal RS (1997). Seminars in medicine of the Beth Israel Deaconess Medical Center. Caloric intake and aging. N Engl J Med.

[4] Canto C, Auwerx J (2009). Caloric restriction, SIRT1 and longevity. Trends Endocrinol Metab.

[5] Lin SJ, Defossez PA, Guarente L (2000). Requirement of NAD and SIR2 for life-span extension by calorie restriction in Saccharomyces cerevisiae. Science.

[6] Rogina B, Helfand SL (2004). Sir2 mediates longevity in the fly through a pathway related to calorie restriction. Proc Natl Acad Sci U S A.

[7] Chen J, Zhou Y, Mueller-Steiner S, Chen LF, Kwon H, Yi S, Mucke L, Gan L (2005). SIRT1 protects against microglia-dependent beta amyloid toxicity through inhibiting NF-kappa B signaling. J Biol Chem.

[8] Lin SJ, Kaeberlein M, Andalis AA, Sturtz LA, Defossez PA, Culotta VC, Fink GR, Guarente L (2002). Calorie restriction extends Saccharomyces cerevisiae lifespan by increasing respiration. Nature.

[9] Anderson RM, Bitterman KJ, Wood JG, Medvedik O, Sinclair DA (2003). Nicotinamide and PNC1 govern lifespan extension by calorie restriction in Saccharomyces cerevisiae. Nature.

[10] Anderson RM, Latorre-Esteves M, Neves AR, Lavu S, Medvedik O, Taylor C, Howitz KT, Santos H, Sinclair DA (2003). Yeast life-span extension by calorie restriction is independent of NAD fluctuation 1. Science.

[11] Cohen HY, Miller C, Bitterman KJ, Wall NR, Hekking B, Kessler B, Howitz KT, Gorospe M, de Cabo R, Sinclair DA (2004). Calorie restriction promotes mammalian cell survival by inducing the SIRT1 deacetylase. Science.

[12] Bordone L, Guarente L (2005). Calorie restriction, SIRT1 and metabolism: understanding longevity. Nat Rev Mol Cell Biol.

[13] Sung B, Park S, Yu BP, Chung HY (2004). Modulation of PPAR in aging, inflammation, and calorie restriction. J Gerontol A Biol Sci.

[14] Lane MA, Ingram DK, Roth GS (1999). Calorie restriction in nonhuman primates: effects on diabetes and cardiovascular disease risk. Toxicol Sci.

[15] Nisoli E, Tonello C, Cardile A, Cozzi V, Bracale R, Tedesco L, Falcone S, Valerio A, Cantoni O, Clementi E, Moncada S, Carruba MO (2005). Calorie restriction promotes mitochondrial biogenesis by inducing the expression of eNOS. Science.

[16] Lopez-Lluch G, Hunt N, Jones B, Zhu M, Jamieson H, Hilmer S, Cascajo MV, Allard J, Ingram DK, Navas P, de Cabo R (2006). Calorie restriction induces mitochondrial biogenesis and bioenergetic efficiency. Proc Natl Acad Sci U S A.

[17] Canto C, Auwerx J (2009). PGC-1alpha, SIRT1 and AMPK, an energy sensing network that controls energy expenditure. Curr Opin Lipidol.

[18] Finkel T, Deng CX, Mostoslavsky R (2009). Recent progress in the biology and physiology of sirtuins. Nature.

[19] Vaquero A, Reinberg D (2009). Calorie restriction and the exercise of chromatin. Genes Dev.

[20] Olgun A (2009). Converting NADH to NAD+ by nicotinamide nucleotide transhydrogenase as a novel strategy against mitochondrial pathologies during aging. Biogerontology.

[21] Jimenez-Hidalgo M, Santos-Ocana C, Padilla S, Villalba JM, Lopez-Lluch G, Martin-Montalvo A, Minor RK, Sinclair DA, de Cabo R, Navas P (2009). NQR1 controls lifespan by regulating the promotion of respiratory metabolism in yeast. Aging Cell.

[22] Villalba JM, Navarro F, Cordoba F, Serrano A, Arroyo A, Crane FL, Navas P (1995). Coenzyme Q reductase from liver plasma membrane: purification and role in trans-plasma-membrane electron transport. Proc Natl Acad Sci U S A.

[23] Navas P, Villalba JM, de Cabo R (2007). The importance of plasma membrane coenzyme Q in aging and stress responses. Mitochondrion 7 Suppl.

[24] Villalba JM, Navas P (2000). Plasma membrane redox system in the control of stress-induced apoptosis. Antioxid Redox Signal.

[25] De Cabo R, Cabello R, Rios M, Lopez-Lluch G, Ingram DK, Lane MA, Navas P (2004). Calorie restriction attenuates age-related alterations in the plasma membrane antioxidant system in rat liver. Exp Gerontol.

[26] Hyun DH, Emerson SS, Jo DG, Mattson MP, de Cabo R (2006). Calorie restriction up-regulates the plasma membrane redox system in brain cells and suppresses oxidative stress during aging. Proc Natl Acad Sci U S A.

[27] Gomez-Diaz C, Villalba JM, Perez-Vicente R, Crane FL, Navas P (1997). Ascorbate stabilization is stimulated in rho(0)HL-60 cells by CoQ10 increase at the plasma membrane. Biochem Biophys Res Commun.

[28] Hyun DH, Hunt ND, Emerson SS, Hernandez JO, Mattson MP, de Cabo R (2007). Up-regulation of plasma membrane-associated redox activities in neuronal cells lacking functional mitochondria. J Neurochem.

[29] Navarro F, Navas P, Burgess JR, Bello RI, De Cabo R, Arroyo A, Villalba JM (1998). Vitamin E and selenium deficiency induces expression of the ubiquinone-dependent antioxidant system at the plasma membrane. FASEB J.

[30] Padilla S, Tran UC, Jimenez-Hidalgo M, Lopez-Martin JM, Martin-Montalvo A, Clarke CF, Navas P, Santos-Ocana C (2009). Hydroxylation of demethoxy-Q6 constitutes a control point in yeast coenzyme Q6 biosynthesis. Cell Mol Life Sci.

[31] Bonawitz ND, Chatenay-Lapointe M, Pan Y, Shadel GS (2007). Reduced TOR signaling extends chronological life span via increased respiration and upregulation of mitochondrial gene expression. Cell Metab.

[32] Smith DL Jr, McClure JM, Matecic M, Smith JS (2007). Calorie restriction extends the chronological lifespan of Saccharomyces cerevisiae independently of the Sirtuins. Aging Cell.

[33] Yang H, Yang T, Baur JA, Perez E, Matsui T, Carmona JJ, Lamming DW, Souza-Pinto NC, Bohr NA, Rosenzweig A, de Cabo R, Sauve AA, Sinclair DA (2007). Nutrient-sensitive mitochondrial NAD+ levels dictate cell survival. Cell.

[34] Reddy VV, Kupfer D, Caspi E (1977). Mechanism of C-5 double bond introduction in the biosynthesis of cholesterol by rat liver microsomes. J Biol Chem.

[35] Keyes SR, Cinti DL (1980). Biochemical properties of cytochrome b5-dependent microsomal fatty acid elongation and identification of products. J Biol Chem.

[36] Passon PG, Hultquist DE (1972). Soluble cytochrome b5 reductase from human erythrocytes. Biochim Biophys Acta.

[37] Jaffé ER (1981). Methemoglobin pathophysiology. Progress Clin Biol Res.

[38] Percy MJ, Lappin TR (2008). Recessive congenital methaemoglobinemia: cytochrome b5 reductase deficiency. Br J Haematol.

[39] Rajcevic U, Petersen K, Knol JC, Loos M, Bougnaud S, Klychnikov O, Li KW, Pham TV, Wang J, Miletic H, Peng Z, Bjerkvig R, Jimenez CR, Niclou SP (2009). iTRAQ-based proteomics profiling reveals increased metabolic activity and cellular cross-talk in angiogenic compared with invasive glioblastoma phenotype. Mol Cell Proteomics.

[40] Price JA, Rogers JV, McDougal JN, Shaw MQ, Reid FM, Kiser RC, Graham JS (2008). Gene expression analysis of bromine-induced burns in porcine skin. Toxicol Lett.

[41] Choudhary C, Kumar C, Gnad F, Nielsen ML, Rehman M, Walther TC, Olsen JV, Mann M (2009). Lysine acetylation targets protein complexes and co-regulates major cellular functions. Science.

